# A machine-learning approach for predicting butyrate production by microbial consortia using metabolic network information

**DOI:** 10.7717/peerj.19296

**Published:** 2025-05-28

**Authors:** Claudia Silva-Andrade, Sergio Hernández, Pedro Saa, Ernesto Perez-Rueda, Daniel Garrido, Alberto J. Martin

**Affiliations:** 1 Programa de Doctorado en Genómica Integrativa, Vicerrectoria de investigación, Universidad Mayor, Santiago, Chile; 2 Laboratorio de Redes Biológicas, Centro Científico y Tecnológico de Excelencia Ciencia & Vida, Fundación Ciencia & Vida, Santiago, Chile; 3 Facultad de Ingeniería, Universidad San Sebastián, Santiago, Chile; 4 Departamento de Ingeniería Química y Bioprocesos, Escuela de Ingeniería, Pontificia Universidad Católica de Chile, Santiago, Chile; 5 Instituto de Ingeniería Matemática y Computacional, Pontificia Universidad Católica de Chile, Santiago, Chile; 6 Instituto de Investigaciones en Matemáticas Aplicadas y en Sistemas, Unidad Académica del Estado de Yucatán, Universidad Nacional Autónoma de México, Mérida, Yucatán, México

**Keywords:** Microbial consortia, Machine learning, Butyrate production, Metabolic network

## Abstract

Understanding the behavior of microbial consortia is crucial for predicting metabolite production by microorganisms. Genome-scale network reconstructions enable the computation of metabolic interactions and specific associations within microbial consortia underpinning the production of different metabolites. In the context of the human gut, butyrate is a central metabolite produced by bacteria that plays a key role within the gut microbiome impacting human health. Despite its importance, there is a lack of computational methods capable of predicting its production as a function of the consortium composition. Here, we present a novel machine-learning approach leveraging automatically generated genome-scale metabolic models to tackle this limitation. Briefly, all consortia made of two up to 13 members from a pool of 19 bacteria with known genomes, including at least one butyrate producer from a pool of three known producer species, were built and their (maximum) *in silico* butyrate production simulated. Using network-derived descriptors from each bacteria, butyrate production by the above consortia was used as training data for various machine learning models. The performance of the algorithms was evaluated using k-fold cross-validation and new experimental data, displaying a Pearson correlation coefficient exceeding 0.75 for the predicted and observed butyrate production in two bacteria consortia. While consortia with more than two bacteria showed generally worse predictions, the best machine-learning models still outperformed predictions from genome-scale metabolic models alone. Overall, this approach provides a valuable tool and framework for probing promising butyrate-producing consortia on a large scale, guiding experimentation, and more importantly, predicting metabolic production by consortia.

## Introduction

Butyrate is a short-chain fatty acid produced mainly by gut bacteria during the fermentation of dietary fibers (Vinolo et al., 2011). This metabolite is an essential factor for maintaining intestinal health, and consequently, the overall health of the human host (Vinolo et al., 2011; Geirnaert et al., 2017; Takahashi et al., 2016; Eslami et al., 2019). More specifically, this metabolite plays a crucial role in the regulation and balance of the gut microbiota, promoting the integrity of the intestinal barrier and reducing inflammation (Tan et al., 2014). In addition to its function as an energy source for colonic epithelial cells (Chen & Vitetta, 2020), butyrate has been shown to have beneficial effects in preventing and treating several intestinal diseases (Cook & Sellin, 1998). The absence of butyrate-producing bacteria species in the gut microbiota is strongly correlated to inflammatory bowel diseases (Geirnaert et al., 2017; Takahashi et al., 2016), and other diseases associated with dysbiosis (Tan et al., 2014; Lan et al., 2024; Coppola et al., 2021; de Alpino et al., 2024). Consequently, understanding the gut microbiome composition and conditions (*e.g*., diet) that can favor its production, is a promising research direction for developing probiotic-based therapies (Kim et al., 2019; Vázquez-Castellanos et al., 2019).

Engineering microbial consortia with desired biological functions is challenging. Design of stable synthetic communities with enhanced productivity for specific metabolites and tailored metabolic functions is complicated due to the uncertainty in the behavior of the members of the community in a given consortium and environmental condition (McCarty & Ledesma-Amaro, 2019; Bernstein & Carlson, 2012; Singh, Ryu & Kim, 2019; Choi et al., 2019; Song et al., 2014; Che & Men, 2019). A common approach for engineering microbial consortia is to assemble different candidate communities *in vitro* and then screen for particular behaviors and functions that promote determined microbial interactions (Choi et al., 2019; Song et al., 2014; Che & Men, 2019; O’Brien, Monk & Palsson, 2015). However, this strategy is very time-consuming and intractable at large scales due to the combinatorial explosion in the experimental design space.

The metabolic behavior of cellular metabolism can be explored using genome-scale network reconstructions (Feist et al., 2009). These representations seek to describe the metabolic potential of the cell by describing the complete repertoire of catalytic functions (enzymes) encoded in the genome (Feist & Palsson, 2008). The reconstruction process typically entails various steps and depends strongly on the amount of available information and intended purpose (Loira, Zhukova & Sherman, 2015; Ebrahim et al., 2013; Thiele & Palsson, 2010). Several tools have been developed to aid in this task, providing the means for the generation of automated reconstructions (Mendoza et al., 2019). Once the network has been assembled, genome-scale metabolic models can be formulated and interrogated with the help of constraint-based modeling methods (Price, Reed & Palsson, 2004). While applications of genome-scale metabolic models are plentiful (Durot, Bourguignon & Schachter, 2009; Feist et al., 2009; Feist & Palsson, 2008; Puchalka et al., 2008; Perez-Garcia, Lear & Singhal, 2016; Mohite et al., 2019; Mendoza et al., 2017; McCloskey, Palsson & Feist, 2013; Pereira et al., 2018), understanding the metabolic network behavior of an organism for increasing the production of metabolites of interest may be regarded as the most prevalent (Che & Men, 2019; O’Brien, Monk & Palsson, 2015).

Genome-scale metabolic model along with constraint-based modeling methods have been particularly useful for exploring and characterizing the interactions within microbial communities (Price, Reed & Palsson, 2004). For instance, MICOM included adjustable dietary constraints and integrated the taxonomic abundance based on metagenomic data to produce personalized metabolic models (Diener, Gibbons & Resendis-Antonio, 2020). The Microbiome Modeling Toolbox employed microbial metabolic reconstructions and metagenomic data as input to model microbiome communities under particular diets (Baldini et al., 2019). The Computation of Microbial Ecosystems in Time and Space (COMETS) was developed to dynamically probe metabolic interactions underpinned by emergent spatio-temporal properties in synthetic communities of up to three members (Harcombe et al., 2014). Notably, these approaches require substantial computational resources for their simulation, which hampers their broad adoption and application to large consortia (Saa et al., 2022). Models, such as OptCom (Zomorrodi & Maranas, 2012) and its extension d-OptCom (Zomorrodi, Islam & Maranas, 2014), focused on optimizing both community biomass and individual growth rates. Methods like Community FBA (cFBA) (Khandelwal et al., 2013) and SteadyCom (Zomorrodi & Maranas, 2012) emphasize maximizing community growth while assuming balanced.

While genome-scale metabolic modeling provides detailed mechanistic insights, simulating metabolic behavior becomes computationally intensive and impractical at large scales due to combinatorial explosion. To address this challenge, machine learning approaches can serve as computationally efficient supplements to genome-scale metabolic modeling, providing rapid predictions that enable researchers to prioritize consortia for further detailed analyses.

In this work, we present a novel machine-learning-based approach informed by automatically generated genome-scale metabolic networks for the prediction of metabolite production by microbial consortia. Briefly, the proposed approach employs machine learning regression algorithms and metabolic network-derived descriptors for capturing the probability of cross-feeding between all pairwise combinations of candidate bacteria. As a proof-of-concept, we evaluated the performance of the approach for the prediction of butyrate production by several consortia of the human gut of up to thirteen members. Butyrate production data from different consortia was simulated using MICOM and then used to train several regressor models that were later validated on actual data. Here, we show how this relatively simple approach achieves satisfactory predictions for butyrate production in different microbial consortia. Most notably, this approach can be readily executed on standard computers once trained, enabling its broad application and adoption by the community.

## Materials and Methods

### Bacterial genomes and metabolic reconstructions

Our analysis included 19 bacterial genomes provided by Dr. Garrido’s laboratory from the human gut microbiota, five *Bacteroides*, two *Phocaeicola*, two *Bifidobacterium*, three *Clostridium*, one *Lachnoclostridium*, one *Escherichia*, one *Flavonifractor*, one *Enterocloster*, two *Lactobacillus*, and one *Mediterraneibacter* (Table S1). Among these, six species produce butyrate: *Clostridium sp. HGF2, Clostridium sp. M62, Ruminococcus gnavus CC55_001C, Clostridium sp. 7_2_43FAA, Flavonifractor plautii 1_3_50AFAA*, and *Llachnoclostridium symbiosum WAL14673* (Gutiérrez & Garrido, 2019). These bacteria were employed to build and simulate all the combinations of synthetic consortia of two up to 13 members with at least one butyrate-producing bacteria. *Clostridium sp. HGF2, Clostridium sp. M62, Ruminococcus gnavus CC55_001C, Clostridium sp. 7_2_43FAA, Flavonifractor plautii 1_3_50AFAA*, and *Lachnoclostridium symbiosum WAL14673* were used as butyrate producer species. Finally, for the computational simulations, automatic metabolic reconstructions were used to build for each bacterial member. Here, the reconstruction tool AuReMe (Aite et al., 2018) was employed using to refer to its closest phylogenetic-related microorganism from the AGORA database (Magnúsdóttir et al., 2017).

### Encoding of metabolic features as vector representation

We used the methodology described by Silva-Andrade et al. (2024) to predict the type of interaction between pairs of bacteria. Briefly, the genome of each bacterium was represented by a fixed-length binary vector, where the presence or absence of each reaction served as a descriptor. The extreme gradient boosting (XGBoost) model (Chen & Guestrin, 2016) was employed to identify and reduce the number of descriptors and their feature rank to select and keep the 25 more important descriptors for each pair of bacteria. These 25 descriptors for each bacteria were next joined into a single vector to describe each consortium. Additionally, we added to this encoding the predicted probability of a cross-feeding for each pair of bacteria in the consortia. The interaction probabilities were derived from the predictor based on the methodology described by Silva-Andrade et al. (2024), which provides a probability value ranging from 0 to 1. Here, a value of 1 represents a 100% probability of cross-feeding interaction, while a value of 0 indicates a 0% probability. These probabilities were encoded by considering all possible pairwise interactions within each consortium. For example, in consortia consisting of three bacteria (A, B, and C), there were three pairs (AB, AC, and BC). There were six combinations in consortia with four bacteria, and this pattern continues up to consortia with 13 bacteria. Each consortium’s encoding thus reflects the predicted interaction probabilities for all possible bacterial pairs within that group using pairwise combinations without replacement.

For each consortium, the amount of butyrate produced was simulated with MICOM (Diener, Gibbons & Resendis-Antonio, 2020) with default parameters, and the mZMB (Medina et al., 2017) medium (described by exchanges in Table S2) as growth medium to simulate the consortia and future validation. In this way, in addition to the butyrate produced by the consortia, two bacteria consortia are described by a 51-feature vector, three bacteria are represented by 78 features, four by 106, and so on.

From the 19 bacteria set, 453,516 consortia containing two to thirteen bacteria were simulated and evaluated, where at least one bacterium was a butyrate producer. The number of examples of consortia created by the number of different members is described in Table 1.

**Table 1:** Simulated consortia with butyrate producer.

Consortium size	Possible combinations
2 bacteria	66
3 bacteria	511
4 bacteria	2,499
5 bacteria	8,210
6 bacteria	21,122
7 bacteria	43,950
8 bacteria	69,063
9 bacteria	88,513
10 bacteria	88,589
11 bacteria	58,236
12 bacteria	45,846
13 bacteria	26,911

### Machine learning algorithms and hyperparameter tuning

A total of 19 microorganisms were selected for training the algorithm. The training set consisted of predicted butyrate production data for 453,516 microbial consortia. To optimize the model’s performance, we employed GridSearchCV from the scikit-learn (Pedregosa et al., 2011) library to fine-tune hyperparameters across several machine-learning algorithms, including Random Forest (RF) regression (Ho, 1995), support vector machine (SVM) (Stephen et al., 2006; Cortes & Vapnik, 1995), XGBoost (Chen & Guestrin, 2016), K-nearest neighbors (KNN) (Zhang, 2016; Yao & Ruzzo, 2006), and Elastic Net (Zou & Hastie, 2005). The dataset was split, with 75% allocated for training and the remaining 25% for testing. K-fold cross-validation was carried out to enhance the models’ robustness.

For KNN, the hyperparameters tuned were n_neighbors ranging from 1 to 21, weights options including ‘uniform’ and ‘distance’, and metric choices between ‘euclidean’ and ‘manhattan’. The RF model was optimized by varying n_estimators between 50 and 200, max_features options (‘sqrt’, ‘log2’), and max_depth ranging from None to 30. For SVM, we explored different values for C (0.1, 1, 10), kernel (‘rbf’, ‘linear’), and gamma (‘scale’, ‘auto’). The ElasticNet model was fine-tuned by adjusting alpha values (0.1, 1, 10, 100) and l1_ratio (0.1, 0.5, 0.7, 1.0). Finally, XGBoost was tuned using n_estimators (50, 100, 200), learning_rate (0.01, 0.1, 0.2), and max_depth (3, 5, 7). The evaluation criterion for hyperparameter tuning was the mean squared error (MSE) on validation sets during K-fold cross-validation. The model with the lowest MSE was selected as the best-performing configuration for each algorithm.

### Prediction performance

To evaluate the prediction performance, we calculated Pearson’s, Spearman’s, and linear correlation coefficients between the predicted and experimental butyrate production values using the Scipy library (Virtanen et al., 2020). Then, the results were visualized using a heatmap, which provided a comprehensive view of the correlation strengths across different models and consortia sizes. Lastly, the best performing model was validated against experimental butyrate production data from consortia composed of two (12 examples), three (10 examples), and thirteen bacteria (10 examples), serving as a blind test to characterize the prediction fidelity. A correlation was considered statistically significant if *p* < 0.05.

### Hyperparameter fine-tuning and model selection

To optimize the performance of our machine learning models, we performed a grid search across various hyperparameters for each algorithm. We tested multiple configurations for ElasticNet, KNN, RF, SVM, and XGBoost models. Table 2 provides an overview of the best parameters found for each model when predicting butyrate production in consortia of two, three, and thirteen bacteria. These parameters were used in the subsequent training and testing phases.

**Table 2:** Selected parameters for ML models.

Consortium size	Models	Parameters
2	ElasticNet	alpha: 0.1, l1_ratio: 0.5
	KNN	metric: ’manhattan’, n_neighbors: 6, weights: uniform
	Random Forest	max_depth: None, max_features: sqrt, n_estimators: 100
	SVM	C: 10, gamma: scale, kernel: linear
	XGBoost	learning_rate: 0.01, max_depth: 3, n_estimators: 200
3	ElasticNet	alpha: 0.1, l1_ratio: 0.1
	KNN	metric: manhattan, n_neighbors: 5, weights: distance
	Random Forest	max_depth: None, max_features: sqrt, n_estimators: 50
	SVM	C: 10, gamma: scale, kernel: linear
	XGBoost	learning_rate: 0.2, max_depth: 5, n_estimators: 100
13	ElasticNet	alpha: 0.1, l1_ratio: 1.0
	KNN	metric: euclidean, n_neighbors: 2, weights: distance
	Random Forest	max_depth: 20, max_features: sqrt, n_estimators: 200
	SVM	C: 10, gamma: scale, kernel: linear
	XGBoost	learning_rate: 0.1, max_depth: 3, n_estimators: 200

### Prediction of butyrate production in simulated and experimental data using regression algorithms

We trained our predictive models by separating the data according to the number of members in the consortia, using K-fold cross-validation to assess the models based on the different consortium sizes. For consortia of two bacteria, the training set achieved a Pearson correlation coefficient of 0.98 with the RF model, indicating a very strong linear relationship. The XGBoost model also performed well in training, with a Pearson correlation of 0.96 and a Spearman correlation of 0.71. For consortia with three bacteria, the KNN model achieved near-perfect correlations in both training and testing, with Pearson and Spearman correlation coefficients of 1.00 and 0.81, respectively, during testing. The XGBoost model also performed exceptionally well in this scenario. In the case of consortia with thirteen bacteria, the testing phase revealed very high Pearson and Spearman correlation coefficients, reaching values of 1.00 in most models, including KNN, RF, and XGBoost. These results indicate that the models were able to capture the relationships effectively, even with the increased complexity of larger consortia.

## Results

### Performance on a validation dataset

We conducted a blind test using actual experimental data from consortia consisting of two, three, and thirteen bacteria. For consortia with two bacteria, the XGBoost (Chen & Guestrin, 2016) model performed the best, achieving a Pearson correlation coefficient of 0.738 (*p* = 0.010), indicating a strong positive linear relationship between the predicted and experimental butyrate production. For consortia with three bacteria, the MICOM (Diener, Gibbons & Resendis-Antonio, 2020) model demonstrated superior performance with a Pearson correlation coefficient of 0.960 ( $p \;< \; 0.001$), reflecting an excellent agreement between the predicted and observed values. In the case of consortia with thirteen bacteria, XGBoost (Chen & Guestrin, 2016) again provided the best performance, with a Pearson correlation coefficient of 0.422, and a Spearman correlation of 0.818 (*p* = 0.004). Although the Pearson correlation was lower compared to smaller consortia, the strong Spearman correlation suggests that the model effectively captured the rank-order relationships despite the increased complexity in larger consortia.

## Discussion

In this study, we present the development of a novel butyrate predictor for bacterial consortia, leveraging metabolic network features and interactions between selected strains. The utilization of metabolic networks has seen a significant rise in recent years. These networks have been employed to enhance the production of specific metabolites (Mohite et al., 2019; Varma, Boesch & Palsson, 1993) and to uncover and comprehend the metabolic characteristics of various microorganisms (Choi et al., 2019; Pereira et al., 2018). Consequently, we created a model integrating bacterial interaction prediction with metabolic network data to forecast butyrate concentrations in diverse bacterial consortia.

**Figure 1:**
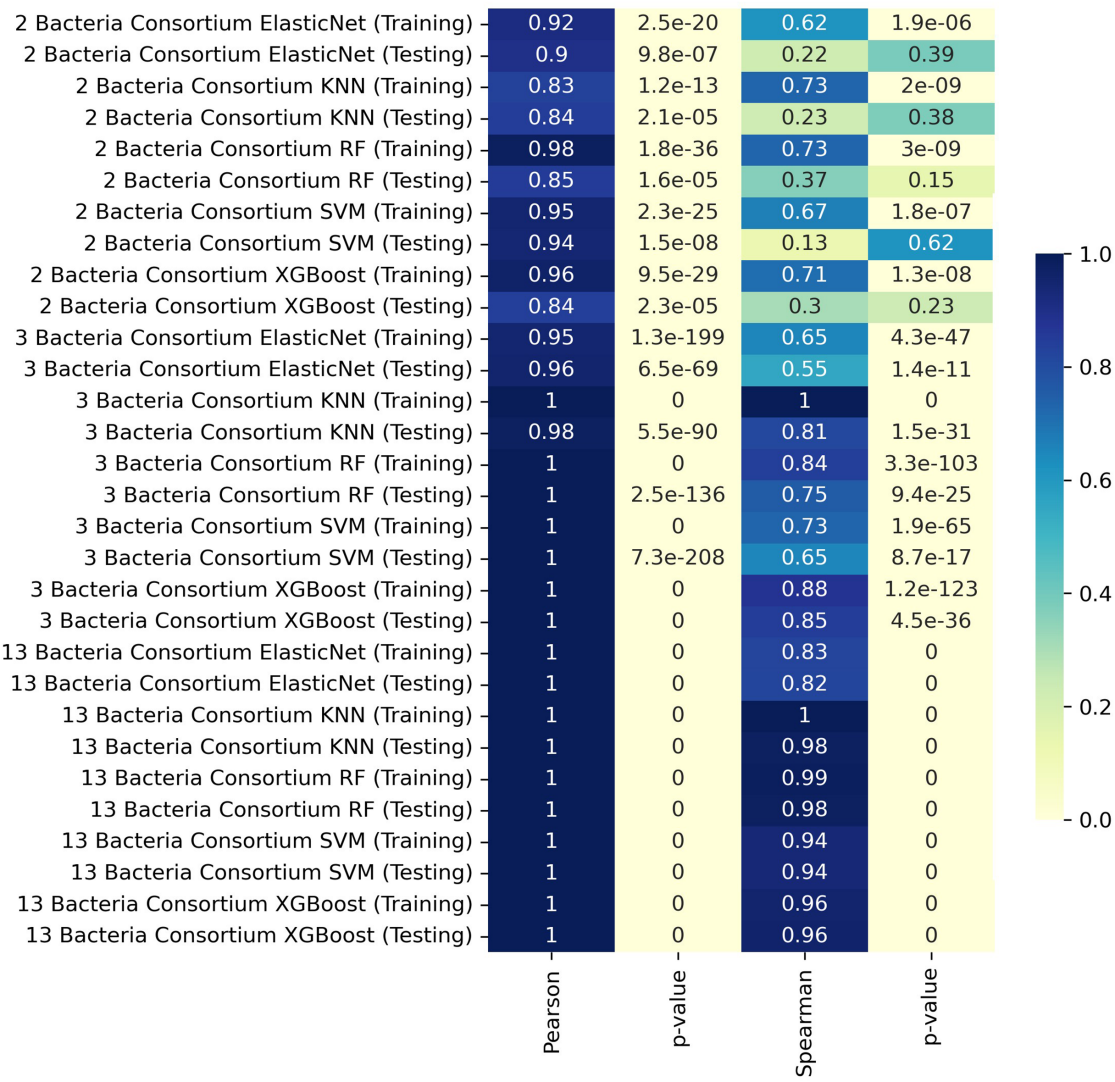
Heatmap representation of Pearson and Spearman and linear correlation in butyrate production (mmol/L) between ML predictions and simulated data.

In some cases, the ML model achieves near-perfect correlation with MICOM-based holdout data, particularly for larger consortia (Fig. 1). This likely occurs because the model learns the specific patterns imposed by uniform abundances and default uptake parameters. However, discrepancies with actual experimental data (where conditions deviate from these assumptions) are expected and highlight the gap between idealized training simulations and complex *in vivo* conditions.

Interestingly, larger consortia often gave higher correlation in the training set, possibly due to multiple butyrate producers being present, dampening any single strain’s variability. In smaller consortia, differences in pairwise interactions or relative abundances may have a stronger effect on butyrate output.

We used a set of 19 gut microbiota bacteria, six of which were butyrate producers. The prediction of butyrate production was evaluated across consortia of different sizes, with varying performance observed across models. For consortia with two bacteria, the XGBoost (Chen & Guestrin, 2016) model outperformed the others, for consortia with three bacteria, the MICOM (Diener, Gibbons & Resendis-Antonio, 2020) model demonstrated superior performance, and in more complex consortia like thirteen bacteria, XGBoost (Chen & Guestrin, 2016) again provided the best performance, effectively capturing the rank-order relationships despite the increased complexity.

For the three-member experimental consortia, MICOM’s predictions correlated better with measured butyrate than our ML models (Table 3). This discrepancy may reflect the sensitivity of small consortia to subtle stoichiometric or ecological factors that are not captured in our training data. The ML models, being trained on a broad uniform-abundance dataset, may have lower accuracy for specific small-scale communities unless further tuned.

**Table 3:** Comparison of model predictions to experimental butyrate production. Values in bold indicate the best-performing models in terms of correlation coefficient and their corresponding *p*-values.

No. of bacteria	Models	Pearson	*p*-value	Spearman	*p*-value	MSE
2	MICOM	0.468	0.147	0.300	0.370	49.408
	ElasticNet	−0.020	0.954	−0.055	0.873	42.675
	KNN	0.043	0.901	−0.196	0.563	35.388
	RF	0.391	0.234	0.218	0.519	49.123
	SVM	−0.870	0.001	−0.973	0.000	54.412
	XGBoost	**0.738**	**0.010**	0.087	0.800	23.441
3	MICOM	**0.960**	**0.000**	0.524	0.120	32.474
	ElasticNet	−0.479	0.161	−0.237	0.510	37.551
	KNN	−0.251	0.484	−0.250	0.486	50.487
	RF	−0.499	0.142	0.061	0.868	95.935
	SVM	0.015	0.966	−0.438	0.206	36.972
	XGBoost	−0.123	0.736	0.365	0.300	56.123
13	MICOM	−0.167	0.645	0.139	0.701	10.172
	ElasticNet	0.153	0.672	−0.034	0.925	14.347
	KNN	0.236	0.511	−0.241	0.503	11.427
	RF	0.086	0.814	−0.139	0.701	9.603
	SVM	0.110	0.763	0.406	0.244	15.530
	XGBoost	0.422	0.225	**0.818**	**0.004**	28.165

These results demonstrate that while MICOM (Diener, Gibbons & Resendis-Antonio, 2020) excels in consortia of three bacteria, the XGBoost (Chen & Guestrin, 2016) model is particularly effective in predicting butyrate production for both smaller (two bacteria) and larger (thirteen bacteria) consortia. This suggests that it is feasible to accurately predict butyrate production in a variety of microbial consortia using these approaches, leveraging interactions between bacteria and the metabolic network information automatically annotated from their genomes.

Other approaches used to design consortia developed different strategies. Some use the abundance of metabolic data and taxonomic information (Diener, Gibbons & Resendis-Antonio, 2020), performed metabolic simulations (Harcombe et al., 2014), or modeled communities using metabolic reconstruction and metagenomic data with a particular diet (Baldini et al., 2019). For instance, Clark et al. (2021) developed a model that determines the contributions of metabolic interactions in the consortium growth and butyrate production.

In contrast to these previously mentioned methods, our approach only uses metabolic network information automatically generated from the annotated genome of microorganisms and the probability of cross-feeding interactions between the pairs of bacteria present in the community, making it the easiest metabolic resource allocation in individual microbes and the least expensive approach available.

The experimentally validated interaction information reported reveals a significant potential for understanding butyrate production between different communities; however, understanding the consortia’s behavior is a significant issue that could be addressed in the future with the help of computational approaches.

A significant limitation of genome-scale metabolic model (GSMM) simulations for microbial consortia is their substantial computational demands, particularly when simulating complex microbial communities. Previous studies have reported computational times ranging from several hours to days when modeling microbial consortia with larger sizes or diverse environmental conditions (Harcombe et al., 2014). In contrast, our machine-learning-based approach significantly reduces computational times; training the predictive models required only a few hours using a server equipped with an Intel® Core™ i9-12900KF processor (24 threads, up to 5.2 GHz) and 128 GB of RAM. Once trained, predictions for thousands of consortia could be performed within seconds, accelerating the screening process compared to traditional GSMM-based methods. It is important to highlight, however, that the exact computational time for training and prediction using ML models may vary depending on each user’s hardware capacity and available computational resources. Nevertheless, even considering such variability, the computational costs associated with our machine-learning approach remain substantially lower compared to GSMM-based strategies.

There is a practical gap in high-throughput screening of candidate microbial consortia for butyrate production: while GSMMs and COBRA methods provide detailed mechanistic insights, they become cumbersome at scale. Our machine-learning framework addresses this need by enabling rapid, approximate predictions for thousands of consortia, which can then be followed by more detailed GSMM simulations on a narrower set of promising candidates.

Although no single ML model universally outperforms all others across every consortium size, XGBoost, Random Forest, and KNN generally provide strong performance. In practice, researchers could select the highest-performing regressor or even adopt an ensemble approach. Critically, these ML models can generate predictions within seconds, considerably faster than running new COBRA simulations for each consortium.

Finally, we must stress that our approach was tested only on the experimental validation. Therefore, there is a possibility of bias in the representativeness of the examples. However, our trained models were suitable for standard computers and could predict butyrate production in a consortium within minutes. In summary, our method, using experimentally validated butyrate producer consortia instead of only simulations, led to promising results that support the idea that our machine-learning approach is encouraging. However, there remains room for improvement and refinement of the method, as more validated information about the consortia that produce butyrate, and their production becomes available.

## Conclusion

The use of metabolic networks to understand microbial consortia behavior has increased over time and, complemented with machine-learning methods, it is possible to increase the speed of data analyses, opening the door to evaluate new methodologies to expand the knowledge about different metabolites that can be produced by a microbial consortium. In this analysis we report a new method to predict the butyrate production in consortia from two to thirteen bacteria, where at least one bacteria of the consortium is a butyrate producer, using a machine learning approach with automatically reconstructed metabolic networks. Despite showing that there is still room for improvement, our method demonstrates an excellent correlation to predict the butyrate production in different consortia.

## Supplemental Information

10.7717/peerj.19296/supp-1 Supplemental Information 1 Supplementary tables.

10.7717/peerj.19296/supp-2 Supplemental Information 2 This framework integrates metabolic network reconstructions and predicted bacterial interactions to generate informative descriptors for microbial consortia. First, metabolic reconstructions are performed for individual bacterial strains, while pairwise interaction probabilities are estimated based on cross-feeding potential. These features are combined into a predictive dataset, which is used to train machine-learning models. The trained models enable accurate prediction of butyrate production, facilitating the rational design and optimization of microbial consortia for targeted metabolite production.

## Additional Information and Declarations

### Data Availability

The following information was supplied regarding data availability:

The data is available at GitHub and figshare:

- https://github.com/networkbiolab/microbial_consortia.

- Silva-Andrade, Claudia (2024). A Machine-learning Approach For Predicting Butyrate Production By Microbial Consortia Using Metabolic Network Information. figshare. Online resource. https://doi.org/10.6084/m9.figshare.27085369.v2.
