# Supplementary material for: A machine-learning approach for predicting butyrate production by microbial consortia using metabolic network information
Source: PeerJ. 2025 May 28;13:e19296. doi: 10.7717/peerj.19296 (PMC12126089; doi:10.7717/peerj.19296)
Supplement: Supplemental Information 1 [file peerj-13-19296-s001.pdf]

Supplementary Table 1. Genus, Strain, and Biosample ID for the Bacterial Genomes

**Genus**	**Strain**	**Biosample ID NCBI**
Bacteroides	*Bacteroides cellulosilyticus CL02T12C19*	SAMN00630422
Bacteroides	*Bacteroides dorei 5_1_36/D4*	SAMN00012892
Bacteroides	*Bacteroides finegoldii CL09T03C10*	SAMN00627907
Bacteroides	*Bacteroides fragilis CL03T12C07*	SAMN16451179
Bacteroides	*Bacteroides ovatus 3_8_47FAA*	SAMN02463791
Bacteroides	*Bacteroides tethaoitamicron VPI 5482*	Sequencing
Bacteroides	*Bacteroides vulgatus ATCC 8482*	SAMN02604309
Bifidobacterium	*Bifidobacterium adolescentis ATCC 15703*	SAMD00061080
Bifidobacterium	*Bifidobacterium longum PT8*	Sequencing
Clostridium	*Clostridium sp. 7_2_43FAA*	SAMN02463700
Clostridium	*Clostridium sp. HGF2*	SAMN00116782
Clostridium	*Clostridium sp. M62/1*	SAMN02415619
Lachnoclostridium	*Clostridium symbiosum WAL14673*	SAMN00110774
Escherichia	*Escherichia coli K-12 MG1655*	SAMD00019998
Flavonifractor	*Flavonifractor plautii 1_3_50AFAA*	SAMN00103468
Enterocloster	*Lachnoclostridium clostridioforme 2_1_49FAA*	SAMN00016629
Lactobacillus	*Lactobacillus paracasei M38*	Sequencing
Lactobacillus	*Lactobacillus plantarum ATCC 8014*	SAMN02463698
Mediterraneibacter	*Ruminococcus gnavus CC55_001C*	SAMN01162086

Supplementary Table 2. Exchange reactions employed for simulating the mZMB medium by the metabolic models.

**Reaction**	**Metabolite**	**Global ID**	**Flux**
EX_4abz_m	4abz_m	EX_4abz(e)	1.000000
EX_ala_L_m	ala_L_m	EX_ala_L(e)	0.251762
EX_asn_L_m	asn_L_m	EX_asn_L(e)	0.225000
EX_asp_L_m	asp_L_m	EX_asp_L(e)	0.571946
EX_cys_L_m	cys_L_m	EX_cys_L(e)	0.035089
EX_gln_L_m	gln_L_m	EX_gln_L(e)	0.820199
EX_glu_L_m	glu_L_m	EX_glu_L(e)	0.820199
EX_gly_m	gly_m	EX_gly(e)	0.157022
EX_h2o_m	h2o_m	EX_h2o(e)	1000.000000
EX_xan_m	xan_m	EX_xan(e)	1.000000
EX_arg_L_m	arg_L_m	EX_arg_L(e)	0.290359
EX_thymd_m	thymd_m	EX_thymd(e)	1.00
EX_gthox_m	gthox_m	EX_gthox(e)	1.00
EX_inost_m	EX_inost_m	EX_inost(e)	1.000000
EX_pnto_R_m	pnto_R_m	EX_pnto_R(e)	1.00
EX_pro_L_m	pro_L_m	EX_pro_L(e)	0.758794
EX_pydx_m	pydx_m	EX_pydx(e)	1.00
EX_sheme_m	sheme_m	EX_sheme(e)	1.00
EX_thm_m	thm_m	EX_thm(e)	1.00
EX_thr_L_m	thr_L_m	EX_thr_L(e)	0.342992
EX_tyr_L_m	tyr_L_m	EX_tyr_L(e)	0.163163
EX_val_L_m	val_L_m	EX_val_L(e)	0.483347
EX_zn2_m	zn2_m	EX_zn2(e)	1.00
EX_h2s_m	h2s_m	EX_h2s(e)	1.00
EX_ribflv_m	ribflv_m	EX_ribflv(e)	1.00
EX_ser_L_m	ser_L_m	EX_ser_L(e)	0.445627
EX_ura_m	ura_m	EX_ura(e)	1.00
EX_trp_L_m	trp_L_m	EX_trp_L(e)	0.092108
EX_ni2_m	EX_ni2_m	EX_ni2(e)	1.000000
EX_h_m	h_m	EX_h(e)	1000.000000
EX_ile_L_m	ile_L_m	EX_ile_L(e)	0.392994
EX_leu_L_m	leu_L_m	EX_leu_L(e)	0.669318
EX_lys_L_m	lys_L_m	EX_lys_L(e)	0.571069
EX_met_L_m	met_L_m	EX_met_L(e)	0.206146
EX_phe_L_m	phe_L_m	EX_phe_L(e)	0.358782
EX_pheme_m	pheme_m	EX_pheme(e)	1.00
EX_pi_m	pi_m	EX_pi(e)	1.00
EX_mn2_m	mn2_m	EX_mn2(e)	1.00
EX_na1_m	na1_m	EX_na1(e)	1.00
EX_nac_m	nac_m	EX_nac(e)	1.00
EX_btn_m	btn_m	EX_btn(e)	1.00
EX_ade_m	ade_m	EX_ade(e)	1.00
EX_mobd_m	mobd_m	EX_mobd(e)	1.00
EX_nh4_m	EX_nh4_m	EX_nh4_m	1.00
EX_ca2_m	ca2_m	EX_ca2(e)	1
EX_cl_m	cl_m	EX_cl(e)	1.00
EX_cobalt2_m	cobalt2_m	EX_cobalt2(e)	1.00
EX_fe2_m	fe2_m	EX_fe2(e)	1.00
EX_fe3_m	fe3_m	EX_fe3(e)	1.00
EX_gua_m	gua_m	EX_gua(e)	1.00
EX_k_m	k_m	EX_k(e)	1.00
EX_mg2_m	mg2_m	EX_mg2(e)	1.00

Supplementary Table 3. Bacteria selected for ML model training

**Code**	**Genus**	**Species**
49FAA	*Enterocloster*	*Lachnoclostridium clostridioforme 2_1_49FAA*
50AFAA	*Flavonifractor*	*Flavonifractor plautii 1_3_50AFAA*
ado_ATCC	*Bifidobacterium*	*Bifidobacterium adolescentis ATCC 15703*
CC55	*Mediterraneibacter*	*Ruminococcus gnavus CC55_001C*
CL02T12c19	*Bacteroides*	*Bacteroides cellulosilyticus CL02T12C19*
Cl03	*Bacteroides*	*Bacteroides fragilis CL03T12C07*
CL09T03C10	*Bacteroides*	*Bacteroides finegoldii CL09T03C10*
D4A	*Bacteroides*	*Bacteroides dorei 5_1_36/D4*
Eco3	*Escherichia*	*Escherichia coli K-12 MG1655*
plant_ATCC	*Lactiplantibacillus*	*Lactobacillus plantarum ATCC 8014*
tet2	*Bacteroides*	*Bacteroides tethaoitamicron VPI 5482*
vul_ATCC	Bacteroides	Bacteroides vulgatus ATCC 8482
w14673	*Lachnoclostridium*	*Clostridium symbiosum WAL14673*
