# Supplementary material for: A machine-learning approach for predicting butyrate production by microbial consortia using metabolic network information
Source: PeerJ. 2025 May 28;13:e19296. doi: 10.7717/peerj.19296 (PMC12126089; doi:10.7717/peerj.19296)
Supplement: Supplemental Information 2 — First, metabolic reconstructions are performed for individual bacterial strains, while pairwise interaction probabilities are estimated based on cross-feeding potential. These features are combined into a predictive dataset, which is used to train machine-learning models. The trained models enable accurate prediction of butyrate production, facilitating the rational design and optimization of microbial consortia for targeted metabolite production. [file peerj-13-19296-s002.pdf]

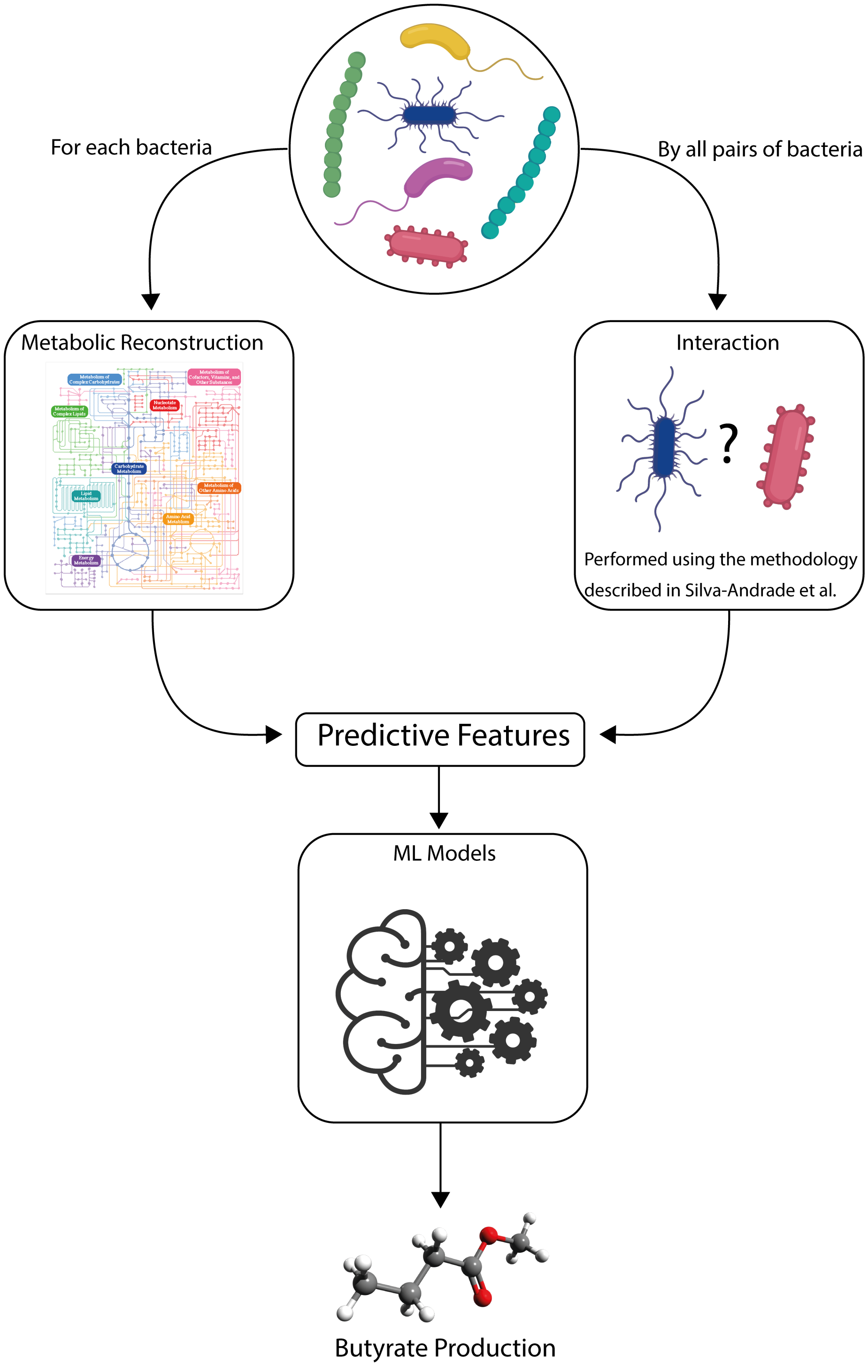

Graphical Abstract: A machine-learning approach for predicting butyrate production by microbial consortia using metabolic network-derived features.

This framework integrates metabolic network reconstructions and predicted bacterial interactions to generate informative descriptors for microbial consortia. First, metabolic reconstructions are performed for individual bacterial strains, while pairwise interaction probabilities are estimated based on cross-feeding potential. These features are combined into a predictive dataset, which is used to train machine-learning models. The trained models enable accurate prediction of butyrate production, facilitating the rational design and optimization of microbial consortia for targeted metabolite production.
